# Follow-Up of Celiac Disease in Adults: “When, What, Who, and Where”

**DOI:** 10.3390/nu15092048

**Published:** 2023-04-24

**Authors:** Chris J. J. Mulder, Luca Elli, Benjamin Lebwohl, Govind K. Makharia, Kamran Rostami, Alberto Rubio-Tapia, Michael Schumann, Jason Tye-Din, Jonas Zeitz, Abdulbaqi Al-Toma

**Affiliations:** 1 Department of Gastroenterology, Amsterdam UMC, Location Vrije Universiteit, 1081 HV Amsterdam, The Netherlands; 2 Center for Prevention and Diagnosis of Celiac Disease, Fondazione IRCCS Ca’ Granda Ospedale Maggiore Policlinico, 20122 Milan, Italy; luca.elli@policlinico.mi.it; 3 Department of Medicine, Celiac Disease Center, Columbia University Medical Center, 180 Fort Washington Avenue, Suite 936, New York, NY 10032, USA; bl114@cumc.columbia.edu; 4 Department of Gastroenterology and Human Nutrition, All India Institute of Medical Sciences, Ansari Nagar, New Delhi 110029, India; govindmakharia@gmail.com; 5 Department of Gastroenterology, Palmerston North Hospital, Palmerston North 4442, New Zealand; kamran.rostami@midcentraldhb.govt.nz; 6 Division of Gastroenterology, Hepatology, and Nutrition, Digestive Disease and Surgery Institute, Cleveland Clinic, Cleveland, OH 44195, USA; rubiota@ccf.org; 7 Department of Gastroenterology, Infectious Diseases, and Rheumatology, Campus Benjamin Franklin, Charité-University Medicine Berlin, 13125 Berlin, Germany; michael.schumann@charite.de; 8 Department of Immunology, The Walter and Eliza Hall Institute, Parkville, VIC 3052, Australia; tyedin@wehi.edu.au; 9 Department of Gastroenterology, The Royal Melbourne Hospital, Parkville, VIC 3050, Australia; 10 Division of Gastroenterology and Hepatology, University Hospital Zurich, University of Zurich, 8091 Zurich, Switzerland; jonas.zeitz@usz.ch; 11 Swiss Celiac Center, Center of Gastroenterology, Clinic Hirslanden, 8032 Zurich, Switzerland; 12 Department of Gastroenterology and Hepatology, St. Antonius Hospital, 3435 CM Nieuwegein, The Netherlands; a.altoma@antoniusziekenhuis.nl

**Keywords:** celiac disease, follow-up, gluten free diet, celiac centers, quality of life, digital platform, digitalized dietary assessment, nutritional education, eHealth

## Abstract

For patients with celiac disease (CeD), a lifelong gluten-free diet is not a voluntary lifestyle choice—it is a necessity. The key end points in clinical follow-up are symptom resolution, the normalization of weight, prevention of overweight, seroconversion, and negation or minimization of increased long-term morbidity. For the latter, a surrogate endpoint is mucosal healing, which means the normalization of histology to Marsh 0–1. Ideally, celiac follow-up care includes a multidisciplinary approach, effective referral processes, improved access that leverages technological advances, and following guidelines with the identification of measurable quality indicators, ideally informed by evidence-based research. Face-to-face CeD care and telemedicine are considered the standards for this process, although published data are insufficient. Guidelines and statements on diagnosis are readily available. However, data are lacking on optimal clinic visit intervals and outcomes and quality indicators such as improvement of symptoms, function and quality of life, survival and disease control, and how to most effectively use healthcare resources. The results of future research should provide the basis for general recommendations for evidence-based standards of quality of care in CeD.

## 1. Introduction

Celiac disease (CeD), an immune-mediated inflammation to gluten (found in wheat, barley, and rye) that affects the small bowel, predisposes patients to malabsorption of nutrients and can cause multiple symptoms, including chronic diarrhea, anemia, short stature, and abdominal discomfort. Over time, it can lead to serious conditions such as osteoporosis, liver disease, neurological disease, depression, and lymphoproliferative malignancy [1]. Celiac disease affects around 1% of the population [2,3]. For the millions of adults and children with CeD, a lifelong gluten-free diet (GFD) is not a voluntary lifestyle choice, it is a necessity, and simply avoiding gluten-containing products after diagnosis is not enough.

Patients, especially in the first years after diagnosis, need expert care and guidance to navigate their dietary and treatment options and manage the disorder as well as related social and nutritional challenges. The majority of CeD patients will need follow-up (FU) with general gastroenterologists, general practitioners (primary or family care), physicians, and dietitians [4,5]. However, follow-up care is inconsistent, inadequate, poorly planned, or absent for many CeD patients [6,7], and 75% of people diagnosed with CeD in childhood do not receive a proper transition to regular follow-ups in adulthood [8]. When celiac patients are followed up on, it has traditionally consisted of routine periodic outpatient clinic reviews, which have, arguably, had limited benefits to the majority of patients’ clinical outcomes. CeD care requires a multidisciplinary approach, and a subgroup of patients may need more dedicated intensive follow-up. There is no clear agreement on guidance on how to organize the systematic follow-up and monitoring of these patients aiming at improving their quality of life and to prevent long-term potential complications. Clear guidelines for a global “standard of follow-up care” are lacking. Appropriate follow-up in developing nations where there are challenges with medical infrastructure and resources remains an unmet need.

To date, the major focus of CeD research has been on epidemiology and diagnosis, with little attention paid to establishing best practices for CeD follow-up and effective models of care [9,10]. A one-size-fits-all follow-up approach strains capacity and increases waiting time. This limits the resources available to provide early intervention to those with persistent symptoms and a deteriorating clinical status and may also impede the focus on the prevention of osteopenia, early detection of associated autoimmune diseases, and downstream social effects of the gluten-free diet.

We aim to give an overview of key issues in the follow-ups of patients with CeD.

## 2. Follow-Up

Much of the research efforts in CeD have centered around pathophysiology, diagnosis, and long-term morbidity. Consequently, there has been less focus on the management and follow-up of patients with CeD, though prior reviews have offered approaches based on expert opinion [1,11,12]. Follow-up is relevant in the long-term, given that persistent symptoms and mucosal changes occur in 20% to 40% of adult CeD patients [13]. There is a lack of observational data about the best logistical approach for the follow up of patients with CeD. Most patients expect to be reviewed on a regular, annual basis through face-to-face follow-up [14,15]. Outpatient in-person clinic review, if necessary, by phone or telemedicine, and prior laboratory tests in their local cities could be an alternative, and even appointments that are made “on request” for patients who are doing well may be sufficient. In our experience, adherence to a GFD is improved with regular FU within the setting of dedicated celiac care. While logically true, whether adherence to a GFD indeed improves quality of life and prevents complications through dedicated celiac care is not known. Celiac-focused outpatient clinics staffed by experienced gastroenterologists as well as expert dieticians may play a critical role in improving adherence to a GFD and preventing or diagnosing early the most serious complications, such as lymphoproliferative malignancies [16,17,18,19]. Most patients now have access to the internet and, thereby, to websites advocating and explaining GFD [20,21]. While this can be advantageous, it also carries the risk that the information is erroneous, incomplete, unsuitable, or confusing.

The key end points in clinical follow-ups are symptom resolution, normalization of weight, prevention of overweight, seroconversion, preventive care (vaccination, screening for osteoporosis, etc.), and negation or minimization of increased long-term morbidity; for the latter, a surrogate endpoint is mucosal healing, which means a resolution of small bowel villous abnormalities (Marsh Grade 0–1) [22,23].

Access to specialized expertise in CeD varies widely worldwide and even within countries. Basic CeD care should be universally available and include:

- a gastroenterologist for diagnosis and medical care;
- a registered celiac dietitian for a supervised gluten-free diet plan and for follow-ups;
- access to a social worker to help with the implementation of the diet at work, school, and in families;
- access to a clinical psychologist for support services.

The care for people with CeD is influenced by patient empowerment, insurance regulations, and the interest of general gastroenterologists in providing FU. It is important to appreciate that 25–40% of patients receive no dietetic input and are often lost to follow-ups in primary care [24,25]. Comprehensive management of CeD requires a coordinated and multidisciplinary team. Ideally, patients are followed up on by a specialist in a dedicated outpatient setting face-to-face or, alternatively, in a telemedical setting [26]. They require monitoring through clinical and dietary reviews, laboratory tests, and, if necessary, endoscopy.

Extra-intestinal manifestations may require discussion in a multidisciplinary team (MDT) involving gastroenterologists, pathologists, dietitians, and, as needed, other specialties such as dermatologists, rheumatologists, psychiatrists, and neurologists [27,28]. The use of prescriptions for osteoporosis, treatments with immunosuppressants, or investigational drugs requires dedicated expertise. Clinicians should be aware of and manage complications, and they should advise the patients regarding the need for long-term monitoring [29,30].

There are limited data on the benefits of screening for associated diseases [31]. Furthermore, no clear outcome measures to set up established Standards of Quality of Care [SQC] for CeD are available. The role of CeD support groups and societies to enhance patient care and follow-up should be recognized, evaluated, and discussed. A key priority is identifying the CeD patients who require more dedicated care in a specialized CeD center to avoid a capacity mismatch and determining what role general practitioners should play in the management of CeD. As the number of gastroenterologists with special interest in CeD is limited, these aspirational goals must be tempered with existing resources.

### 2.1. Disease Monitoring (When, What, and Who?)

Current recommendations in the guidelines [29] by the European Society for the Study of Celiac Disease (ESsCD) emphasize systematic follow-up care for those diagnosed with CeD, which is consistent with follow-up proposed by the new ACG guidelines [30]. Routine assessment should evaluate GFD adherence, determine nutritional status, and monitor for complications based on a combination of history, CeD serology, and other lab-based tests. A CeD dietitian with GFD expertise is important for providing GFD education and reviewing its progress, including the adequacy of GFD knowledge and practices. Monitoring should include verification of the normalization of laboratory abnormalities detected during follow-up.

An unanswered question from the available literature is when and how often patients with uncomplicated CeD should be monitored [29]. Regardless of the provider, long-term monitoring, including, at least, a two-year FU interval in stable patients is universally encouraged. In between, FU by GPs can be considered in some specific situations.

Several key points in follow-up should be noted:

1. Recognition of “slow responders”, which are CeD patients who still report symptoms six months to a year after initiation of a GFD. This is a common occurrence, and while some consider these patients to be “non-responsive‘’, a cause can usually be identified when a systematic approach to follow-up is undertaken. The most common cause is an ongoing gluten intake, albeit unintentional.
2. Increased awareness of neurological symptoms that are related to gluten, including gluten ataxia, peripheral neuropathy, foggy mind, anxiety, and depression [32].
3. Consultation is needed with a dermatologist when there is a suspicion of dermatitis herpetiformis (DH), the skin manifestation of CeD [33].
4. Monitoring of people with CeD should include verification of the normalization of laboratory abnormalities detected during the initial laboratory investigation. Upper gastrointestinal (GI) endoscopy with duodenal biopsies is recommended for monitoring in cases with a lack of clinical response or relapse of symptoms despite a GFD [29,30,34].
5. Metabolic syndrome and fatty liver disease should be monitored in CeD patients [35]. Abnormal liver function tests are a common finding in CeD, with the strongest association reported at presentation or diagnosis. CeD hepatitis is manifested by mild hypertransaminasemia (three to five times the upper limit of normal) and is due to a gluten-dependent liver injury that settles on a GFD [36,37]. Autoimmune liver diseases such as autoimmune hepatitis, primary biliary cholangitis, and primary sclerosing cholangitis are also more common in celiac disease. An increasingly reported complication is that of non-alcoholic fatty liver disease, which can occur as part of metabolic syndrome after starting the GFD. Long-term GFD has been associated with metabolic dysregulation and cardiovascular complications. Patients with metabolic dysfunction-associated fatty liver disease need strict counseling regarding increasing physical activity and optimizing their diet to reduce caloric intake, enrich unprocessed, naturally gluten-free foods, and minimize highly refined carbohydrates and saturated fat [38,39,40].
6. The timing of bone density studies should follow a CeD-specific schedule with a defined age to start DXA screening [29]. It is important because osteopenia and, less frequently, osteoporosis are common in CeD. This applies for both females and males.
7. Associated auto-immune conditions (in particular hypothyroidism) need to be checked regularly [28].
8. Some CeD patients, especially young adults at diagnosis, have an increasing need for psychosocial counseling [41].
9. CeD is associated with an increased risk of pneumococcal sepsis and mortality, and, therefore, pneumococcal vaccination is recommended, although practices differ widely. Some guidelines (ACG) recommend this vaccine for all adults with CeD [30], but, in some centers, it is given arbitrarily to all CeD patients with smaller spleens (125 cc) or beginning at the age of 70 years [42]. More generally, vaccination schedules for various infectious agents should be clarified for people with CeD.
10. Tissue transglutaminase antibodies (IgA anti-TG2) have been shown in published studies to be insufficient for predicting relapse; other biomarkers are required, similar to the impact of fecal calprotectin measurement in the care of IBD patients [43].
11. Proper data are still lacking on long-term outcomes in follow-up disease activity scores, such as the frequency of outpatient visits, histological follow-up, and cost effectiveness. CeD guidelines suggest that a full assessment of disease activity, such as antibodies and investigating for deficiency of essential elements, needs to be performed before starting GFD and after an adequate period, e.g., 12–24 months, to assess reversal or improvement in the manifestations of CeD [29,30].
12. Specific attention to the possibility of refractory CeD (RCD); making the distinction between RCD type I and type II; and closely monitoring the nutritional status of both types. Clinicians require a “Red Flags” index for the diagnosis of refractory CeD, which carries a higher risk of developing lymphoproliferative malignancies [1,44]. A simple “Red Flags” index, using early signs and symptoms for family members and high-risk patients for developing CeD, might be useful. Specialist care of refractory CeD is important, and expediting review by an appropriate specialist may be aided by such a tool. It is recommended that patients with RCD II should be referred to secondary celiac centers with RCD-experienced gastroenterologists, immunologists, and hematologists [29].

Table 1 shows a suggested scheme for CeD patient follow-up. 

### 2.2. Assessment of Dietary Adherence during the Follow-Up

Adherence to a lifelong, strict GFD is necessary to induce CeD remission, aiming at improving the quality of life, controlling symptoms, correcting nutritional deficiencies, and minimizing the risk of long-term complications [45]. However, a significant percentage of patients have difficulty maintaining dietary restrictions [46]. Currently, the available methods to assess adherence to GFD are: clinician and dietitian interviews and measuring CeD-specific antibody titers. Performing a follow-up duodenal biopsy is another, less favored option and recommended only in certain clinical scenarios, such as those with persistent or recurrent symptoms without other explanations [29].

However, these approaches are not sufficiently sensitive. It is well known that negative anti-TG2 antibodies do not necessarily indicate good GFD adherence and also poorly correlate with mucosal recovery [47,48,49]. Thus, a combination of these methods is needed to improve the efficacy of dietary evaluation. However, new laboratory tests to monitor adherence are still awaited in clinical practice, such as the detection of gluten immunogenic peptides in feces [50] or intestinal fatty acid binding protein (I–FABP) in the blood [51].

Dietary assessment remains central in this process. It should be performed by qualified dietitians with appropriate training in CeD. There are different methods in use, such as food diaries, 24 h recalls, dietitian interviews using short questions, self-reported questionnaires, or food frequency questionnaires. However, there is a need for more objective and standardized tools of assessment [52,53].

Biagi et al. proposed a scoring system based on four simple questions that could be used even by nonexpert personnel. This system classified patients into three groups: those who do not follow a strict GFD; those who follow a GFD but with significant mistakes that need correction; and those who follow a strict GFD [52]. Two other methods were proposed by Leffler et al. [54]. The first was a Standardized Dietician Evaluation (SDE) based on a detailed interview conducted by an experienced dietitian. The second tool, Celiac Dietary Adherence Test (CDAT), was less time-consuming and could easily identify patients at high risk of poor adherence. Both methods were validated and allowed for a reliable assessment of gluten exposure.

### 2.3. Nutritional Education

It can be difficult to completely avoid all gluten-containing foods, and adherence to a GFD varies between 42% and 91%, depending on the population studied [55]. Suboptimal adherence may relate to a variety of demographic, psychosocial, and clinical factors [56,57,58]. Hurdles to a strict adherence to GFD include insufficient awareness and education of patients concerning their disease, sources of food contamination, and the inadequacy or lack of information on packaged food labels [59].

Awareness of what a GFD implies is of great clinical significance because misinterpretations concerning the gluten content of foods can lead to unrecognized gluten exposure and, subsequently, persistent mucosal damage [55].

The degree of adherence is higher in those patients with a higher education; this may be due to the fact that well-educated patients might have a better perception of their disease and its treatment and, consequently, are more adherent [60].

Nowadays, it is easier to obtain information on gluten-free products [61]. People search for information themselves without consulting a health care professional, and this may lead them to receive a more complete picture of CeD in general. At the same time, this depends on the reliability of the available information.

For patients with CeD to stay healthy, they need to know how to manage a lifelong diet, where to obtain cost-effective foods, and how to reduce the risk of cross-contamination. It is, therefore, vital to inform patients and convince them of the benefits of a controlled diet. To promote adherence, self-management programs, which include education, behavioral strategies, and combinations thereof in the form of multidisciplinary care programs, are needed [62,63].

Patients are more motivated to adhere to a GFD when they have the perception that the healthcare providers communicate well with them and actively encourage them to be involved in their own care. Supporting and informing patients should be an integral part of the follow-up of patients with CeD.

### 2.4. Indications for Follow-Up Small Bowel Biopsy after GFD

In adults, serum IgA anti-TG2 titers have poor sensitivity for predicting persistent mucosal architectural distortion [49]. In contrast, a high titer of anti-TG2 antibodies has been reported to be associated with severe mucosal changes, to the extent that no initial biopsy approach has been advocated for in a subgroup of children and, potentially, adults.

Performing an early routine biopsy (at 6 months) has been proven in numerous studies to be unhelpful. A degree of villous atrophy is present in about 40% of patients who are re-biopsied at 12 months, despite good dietary compliance [64,65,66]. There is insufficient evidence to support a mandatory follow-up biopsy as a routine in asymptomatic patients.

Current data suggest that a personalized follow-up approach is needed, wherein follow-up biopsies are performed only for a selected group based on age, initial disease severity, and response to the GFD [65]. Biopsy is needed in those with persistent symptoms despite adopting a strict GFD or in patients who develop additional red flag symptoms. A biopsy examination, preferably in combination with T-cell flow cytometry, is needed in those with persistent symptoms despite adopting a strict GFD or in patients who develop additional red flag symptoms. In these patients, refractory celiac disease should be excluded [67,68].

It seems wise to do a follow-up biopsy in adults with a severe initial presentation, especially those older than 40 years, after 1–2 years of starting a GFD to assess for mucosal healing. Furthermore, a follow-up biopsy is the only way possible to confirm a response to GFD in patients with seronegative celiac disease.

### 2.5. Screening for Celiac Disease in Family Members

Screening for CeD in family members is a major topic discussed during an FU. Patients realize that it might be beneficial because it can run in families. First-degree relatives of CeD patients have a pooled prevalence of 7.5%. The prevalence of CeD among first-degree relatives varies substantially with their specific relationship with the index patient, with sisters and daughters at the highest risk (1 in 7 and 1 in 8, respectively) [69]. In general, it is recommended to screen symptomatic first-degree relatives [29]; screening asymptomatic family members is not recommended but may be considered [70].

The strong association between CeD and specific human leukocyte antigen (HLA) genes makes HLA genotyping a useful tool in specific situations. The main susceptibility genes (HLA-DQ2, specifically HLA-DQ2.5, HLA-DQ2.2, and HLA-DQ8) are found in almost 99% of CeD patients, compared with 20–40% in control populations [71]. The frequency of these genes is higher in family members with CeD than in the general population. Although these genes, especially HLA-DQ2.5, impart a substantial relative risk for CeD, the absolute risk is low. HLA typing is a “once-only” test that is required in family screening once in a lifetime. A relative without HLA susceptibility does not require further monitoring for possible CeD.

The next step in screening is a blood test for the IgA-TG2 antibody. If this is positive, then histological examination of duodenal biopsies is recommended. If it is negative, the primary care physician or physician should repeat the test; however, the optimal interval for re-testing has not been defined. Rescreening after 5–10 years benefits relatives [72], underscoring that the incidence of CeD is increasing over time [73].

### 2.6. Interdisciplinary Team Membership

While care delivered by a MDT in a secondary CeD center, guided by best-practice guidelines, might be mandatory to offer an effective model for long-term follow-up of patients with CeD, research on cost effectiveness in relation to specialist CeD services is limited [74].

This might also be organized as part of a service specializing in chronic GI inflammation and benefiting from knowledge on managing autoimmune diseases [28]. As such, it might become the favored model of healthcare delivery in CeD among healthcare practitioners supported by insurance companies. Recommendations for personnel included as members of such CeD centers should be discussed, evaluated, and reported. If this team is part of or has an overlap with a multidisciplinary clinic team providing care for chronic inflammatory patients that have similar overlapping problems such as additional autoimmune skin manifestations, arthritis, vasculitis, or autoimmune liver manifestations such as autoimmune hepatitis, primary biliary cirrhosis or primary sclerosing cholangitis, a dermatologist, a rheumatologist, and a hepatologist might be included [27]. Other potentially valuable members of the core team might include psychologists, as mental health issues such as anxiety and depression are common in CeD and can impact quality of life and dietary adherence [75,76].

The clinical phenotype of CeD is highly heterogeneous [77]. Consequently, there is significant variation in the pattern and complexity of symptoms and associated medical issues between patients and over time even in the same patient; as such, the care of patients with celiac disease requires flexibility and individualized clinical management with collaboration between the patient and members of such a team. On the other hand, specifically trained dietitians might be able to provide effective follow-up for CeD patients who are performing well on their GFD. In some centers, they are empowered to order specific laboratory tests to confirm the reversal of deficiencies and the effectiveness of the diet, and the interpretation of such tests does not necessarily require the presence of a CeD specialist. This has some similarity with the management of patients suffering from Crohn’s disease and ulcerative colitis, as they are frequently seen by nurse practitioners in Inflammatory Bowel Disease units [78].

General practitioners (GPs) and other primary care providers may play an important role in family screening and FUs of previously diagnosed CeD patients; however, their ability to effectively do this will depend on patient load. GPs will be a solution if the critical number of diagnosed celiacs in a local population is high enough, such as in Finland [79]. However, GPs nowadays are burdened with increasing workloads in various western countries [80]. This might be a particular problem for CeD patients, who need additional time to have their health issues addressed. Then, again, a minimal critical number of CeD patients per GP is required; otherwise, GPs are not yet a beneficial option for the majority of CeD patients for FU as their medical experience, without sufficient guidance, as these will be inadequate to manage specific CeD needs.

### 2.7. Celiac Disease Center and Coordinated Care Models

To determine if patients need specific care at a secondary CeD center, a triaging gastroenterologist should be available to review the medical history and recommend tests if necessary. For the diagnosis of CeD, there might be a misuse of diagnostic tests [81], which has been observed in both GPs and gastroenterologists. Given the number of people who have an indication for testing, this should be performed not only in CeD centers but in many health care settings.

**Figure 1:**
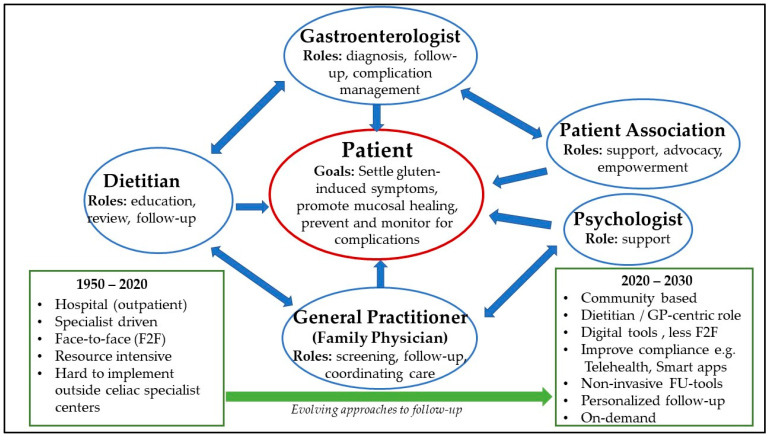
Evolving approaches to CeD follow-up.

Several integrated care models for CeD have been proposed, but detailed clinical pathways and outcome improvement evaluations are needed. Figure 1 illustrates evolving approaches to CeD follow-ups. Initiatives in CeD begin with the development of quality measures but lack clear clinical pathways [82].

CeD patients with a clinically inactive disease and a GFD might delay their outpatient clinic visits or do not follow-up. The evaluation of such reduced care is not yet available.

A limited number of formal CeD centers have been established in the USA, Europe, India, and Australia. These are generally led by a gastroenterologist with a specially trained CeD dietitian and include services such as a weekly medical clinic, a telephone helpline staffed by a dietitian or nurse practitioner, scheduled phone follow-up, active nurse management of appointments, written educational information leaflets, and online resources [83,84,85,86].

Original research that measures educational actions on documented adherence to quality measures in CeD should be undertaken and used to support evidence of structured actions aimed at quality improvement. Data on improvements in outcomes, cost savings, and patient-reported outcomes as a result of such an attitude are required. Data mining by insurance companies should be discussed and ethically considered. Implementing and standardizing CeD care has the potential to improve the quality of care and health outcomes for patients.

### 2.8. Celiac Disease Patient Registries

While a patient registry is considered mandatory for a secondary CeD center, it is a challenging task to initiate and maintain. International CeD societies should consider supporting the framework for CeD registries. Recognizing the time, cost, and efforts involved in maintaining such registries is mandatory. National registry initiatives exist in several countries and may provide access to resources that otherwise might be too challenging for local CeD centers [87,88]. Such registries would be invaluable sources of information for better understanding how patients are diagnosed and followed up on as well as for designing better approaches. A CeD smart phone app that transfers clinical information from the patient directly to the caretaker in the CeD center, as it is frequently performed with PROs in clinical studies, might be a bridge for registries to import data and to contact patients when lab or personal contact is indicated. However, the impact of privacy regulations on data mining would need to be considered.

## 3. The Setting of Clinics

### 3.1. Face-to-Face

Management of CeD patients has historically consisted of routine periodic clinic reviews, which, arguably, have limited benefit for the clinical outcomes of patients who are already stable. This utilizes capacity and increases waiting list times for those who need special attention. This also limits the resources available to provide early intervention to patients whose condition is deteriorating, resulting in poorer patient outcomes and preventable hospital admissions. Stretched staff resources cause long waiting times for outpatient care, resulting in adverse patient outcomes for patients who need more urgent care. The use of routine appointments results in stable patients being reviewed without the true necessity of a face-to-face consultant appointment. This adds to the waiting list with little benefit to individual patient outcomes.

Outpatient clinics should be adaptable to longer wait times. This needs to be performed in a way that maximizes the use of resources in order to improve the quality of care that can be delivered to patients. It is important to consider redesigning outpatient clinics to provide open access through the telephone, telemedicine, secure phone messaging services, and email support. Most US-based systems advocate for patient portals and not email-given privacy concerns. These digital tools should be applied in accordance with local privacy policies. These advancements allow patients to become educated on how to monitor their condition, allowing them to detect deterioration and seek professional help. Patients who are stable but have concerns can be reassured without the need for a face-to-face appointment.

### 3.2. Virtual Clinics and Dietitians in the Lead

The COVID-19 epidemic helped us to develop and improve virtual outpatient clinics [78]. Web-guided care has rapidly developed during the COVID-19 pandemic (2020–2023) and should be evaluated to determine if it benefits patients and has a positive impact on their quality of life and adherence to GFD. In the Netherlands, the telemedicine system “myIBD coach” for inflammatory bowel disease patients has been shown to be safe and to reduce outpatient visits and hospitalizations when compared to standard care [89]. Similar outcome studies in CeD are urgently needed.

To monitor CeD patients through smartphone applications, celiac centers might develop and validate scoring systems for this [90]. A CeD-Monitoring Index (consisting solely of patient-reported outcomes) might reduce time to recovery, increase GFD adherence, and reduce the number of F2F-outpatient visits. Studies are needed to assess whether the course of CeD on the GFD can be improved.

Digital platforms could practically be a good tool for follow-up of CeD patients, provided that they are easy to use, including downloads on electronic devices used daily. However, there is, as yet, no specific dietary software for CeD. One article discussed the use of one platform, which provides an eHealth tool for assessing the dietary profile and other health parameters of celiac patients, for example, body composition, biochemical data, or symptomatology [91]. This platform can be used by both health professionals and celiac patients interested in their nutritional status and dietary pattern.

Gradually, videocalls are becoming the standard of care with high patient satisfaction due to easy scheduling, increased flexibility, and shorter wait and/or travel times. Regular follow-up via a telephone-based approach has been associated with improved dietary adherence [91,92]. Future research to explore the optimal use of these non-F2F technologies to enhance follow-up is warranted.

CeD patients with stable disease under GFD should be evaluated every 12–24 months in collaboration with GPs, a specialized nurse or dietitian reviewing blood tests, and online questionnaires for follow-up [93]. Dietitians work with patients to develop comprehensive, personalized nutritional plans focused on restoring health and managing the symptoms of celiac disease on a daily basis. Dietitians with the ability to order laboratory tests would strengthen their care and the overall CeD care at FU.

### 3.3. General Practitioners’ Key Roles in the Future

CeD is becoming one of the most prevalent autoimmune diseases encountered in general practice, and general practitioners (GPs) might develop a central role in its work-up for diagnosis and follow-up if a critical number of patients per GP are diagnosed. Due to the fact that these critical numbers have not yet met in the majority of counties, possibly only with the exception of Finland, this should be reconsidered in the future. Given the time pressures on hospital-based gastroenterologists and dietitians, GPs could be empowered to take a key role in follow-up, but it will be important to ensure they are provided education, consensus guidelines to inform best practices, and have ready access to specialist input from a celiac-specializing dietitian and gastroenterologist [14,25].

Key challenges are improving the current poor rate of detection by GPs, distinguishing it from non-celiac gluten sensitivity, and monitoring and optimizing treatment to enhance long-term outcomes.

### 3.4. Patient Preferences

A UK study suggested FU by a dietitian with a doctor available or a hospital doctor was preferred over a GP follow-up, a lone dietitian follow-up, no follow-up, or access when needed [Personal communication, Unpublished data]. Follow-ups were found to be useful for general reassurance as well as for the review of symptoms and CeD serology. Notably, most patients were willing to accept follow-ups by non-traditional methods such as telephone or video reviews. The study highlighted age-related differences in preferences for follow-up, with non-traditional approaches more favored in the younger cohort (18–25 years vs. >66 years old). This is likely to represent higher levels of digital literacy and familiarity in the younger population.

Further supporting the value of non-traditional F2F approaches are studies showing that a GP and nurse practitioner-led telephone-based strategy can achieve many of the recommended follow-up requirements and that proactive contacting of patients ensures stronger engagement [14]. Regular follow-up improves dietary adherence even when utilizing a telephone-based approach [7,92].

A Swedish study showed long-term care provided by a GP or gastroenterologist produces similar outcomes based on laboratory variables and physical and mental health scores. However, patients in the general practitioner group had lower dietary adherence and were less likely to have seen a dietitian initially or at FU, suggesting a need to highlight the key importance of dietitians in CeD follow-ups to GPs [15]. In Finland, most celiac FUs are similarly undertaken in primary care, and attaining good adherence is achievable [61].

As non-traditional approaches, including phone and telehealth consultations, are likely to continue in light of the COVID-19 pandemic, and further research is crucial to understand the implementation, benefits, and limitations of these approaches and which subsets of CeD patients may benefit the most.

## 4. Gluten-Free Food in Outpatient Clinics and during Admission to Hospitals

If CeD patients have to spend time in a hospital, whether it is for overnight care, an emergency visit, or an outpatient clinic visit, they know how difficult it can be to have proper access to a GFD [94]. Most hospital cafeterias are not equipped to handle cross-contamination. A dietitian needs advanced notice before a CeD patient arrives to notify and prepare their chefs and kitchen staff with appropriate directions in case of hospitalization. Nevertheless, patients with CeD should expect to have a diet in a hospital free of cross-contamination. CeD patients should be able to bring their own prepared foods and store them appropriately in their ward’s pantry or refrigerator during hospitalization. As CeD diagnosis has been on the rise in recent decades, hospitals should organize safe options, and any hospital admission should include a mention of a co-diagnosis.

Having gluten-free options such as fresh fruit, vegetables, eggs, and nuts should be the easiest food items to keep in stock at the outpatient clinics of celiac centers and, also, at daycare centers in general [95]. Hospital management catering should think outside the box of traditional foods such as gluten (wheat), corn, and soy. Hospital trusts should defined a specific pathway for patients with CeD while in an outpatient clinic or hospital [94,95].

## 5. Conclusions 

The comprehensive management of CeD requires a strategic approach and dedicated teams. Although both providers and patients are convinced that a well-structured CeD unit with dedicated personnel and a multi-disciplinary approach that aims to achieve remission and restore a good quality of life is the best way to manage CeD, the evidence supporting such an approach remains limited.

The current challenge of managing CeD is ensuring the success of the GFD. This explains the key role of the dietitian during the process of its implementation and follow-up. Gluten is also frequently used in the solid phase of many forms of drugs, especially generic drugs, which requires awareness from physicians, and especially from the pharmacist when delivering drugs and justifies the valuable contribution of the pharmacist in the overall management of CeD patients. Regulatory boards for drug registration request that major studies reregister gluten-free generics on the market.

A lifelong GFD is the only proven treatment for CeD. Small amounts of gluten can trigger symptoms and damage the bowel mucosa [96]. We recommend establishing a GFD masterplan under the supervision of a specialized celiac GFD dietician, who can teach the patient about great-tasting products, what ingredients to look for on labels, shopping tips, and how to choose meals at restaurants. Different tools are available to monitor GFD in the absence of expert gluten free dieticians. Dedicated questionnaires could be used to evaluate knowledge and attention toward the gluten-free attitude of the patients [52,54]. More recently, direct tools based on the detection of gluten immunogenic peptides in stool and urine have been introduced to monitor GFD and inadvertent gluten ingestions; in this case, the amount and frequency of gluten exposure might help identify patients without symptomatic and/or histological normalization, with persistently negative tests, or those who have achieved mucosal healing [97]. The role and accuracy of these tools require more elaboration before they can be implemented in daily clinical practice.

About 80 to 90 percent of people who follow a GFD notice an improvement in their symptoms after a few weeks to a few months. However, healing of the small bowel may take months or years. When it does heal, inflammation of the intestine subsides, and it can absorb nutrients from food.

With a GFD, elevated CeD antibodies (IgA-TG2 antibodies) start to trend down. To monitor responses to a GFD, we recommend checking these antibodies every six months until they return to normal. For those who continue to have symptoms, a follow-up endoscopy to monitor mucosal healing may be performed [30].

Additional support is sometimes warranted if there is no improvement with a GFD. Typically, this could be caused by small amounts of gluten still being consumed from hidden sources, such as modified food starch, preservatives, and stabilizers made with wheat. Other food products, such as corn and rice products, may be contaminated with wheat gluten if they are produced in factories that also manufacture wheat products.

Other food products, including corn and rice products, are produced in factories that also manufacture wheat products and may be contaminated with wheat gluten.

The primary medical care in Europe is not different from that in the USA, Australia, or India in this respect. Children and adults have their own general practitioners, but few of these have an acceptable knowledge of CeD or its follow-ups. Unfortunately, a significant number of patients (up to 30% or more) are lost to follow-ups [4,6,7,25]. There are not enough clinicians with a dedicated interest in CeD; most patients are probably checked into non-dedicated general gastroenterology clinics by doctors who have a variable knowledge of or interest in CeD.

Elderly CeD patients who have a bone fracture face a serious yet potentially preventable risk of breaking another, often within the next two years. This is especially true for people 65 and older who break a hip or who develop a spinal fracture [98]. These secondary fractures can result in life-limiting disabilities and a permanent loss of independence. One in five patients die within a year of surgery for a hip fracture. Yet, those at risk of a repeat fracture often receive inadequate follow-ups. After their broken bones have healed, far too few patients are referred for parenteral treatments that could stave off another costly, debilitating, and, sometimes, deadly fracture.

CD is associated with an increased risk of psychosocial problems, such as depression, anxiety, and eating disorders [99]. Social implications of the GFD (social isolation, avoiding going out because of the risk of contamination, etc.) are usually blamed for this. Psychological support is necessary and may improve acceptance and subsequent adherence to the GFD, as well as reducing the risk of anxiety and depression [100]. Therefore, psycho-social professionals (such as a psychologist, social worker, and others) should be an integral part of the multi-disciplinary follow-up team.

In summary, CeD follow-up aims to optimize patient outcomes by addressing symptom resolution, complication monitoring, and the minimization of long-term morbidity through mucosal healing. There is a paucity of data to inform the optimal approach to achieving these outcomes, and the follow-up of patients with CeD is inconsistent and widely variable. There is a need for the development of models of care for patients with CeD that facilitate effective, accessible, affordable, and quality-driven care for everybody.

### Key Points

○ Follow-ups of patients with CeD are important and include ensuring symptom resolution, optimization of nutrition and weight, normalization of serology, nutrient levels and bone density, preventive care, and minimization of long-term morbidity.
○ Dieticians as well as psychosocial professionals should be an integral part of the multi-disciplinary follow-up team.
○ CeD patient follow-ups are inconsistent and variable, and more studies are needed to inform on the best approach.
○ An important surrogate endpoint of progress on a GFD is small bowel mucosal healing.
○ There is a requirement for a “Red Flags” index for the diagnosis of refractory CeD, which carries a higher risk of developing lymphoproliferative malignancies.
○ RCD II should be referred to secondary CeD centers with RCD-experienced gastroenterologists, immunologists, and hematologists.
○ GPs will be a solution if the critical number of diagnosed celiacs in a local population is high enough.
○ There is a need for models of care for CeD patients that facilitate effective follow-up and utilize health care resources in an efficient manner; the use of technologies such as video calls and smart phone apps carry a lot of appeal, but more research is needed.
○ A CeD-Monitoring Index (consisting solely of patient-reported outcomes) might reduce time to recovery, increase GFD adherence, and reduce the number of F2F-outpatient visits.

## Figures and Tables

**Table 1:** Suggested follow up scheme for adult CeD, modified from Al-Toma et al. [29].

Time	What Is Needed	Who *? How?
At diagnosis	Physical Examination (BMI)	Physician and dietician. Face-to-face
	Counselling by a “celiac” dietician	
	Discuss family screening	
	Recommend Celiac Society or Support group	
	Serology (IgA-anti TG2), lab, DXA (30–35 years start) or at diagnosis in special scenarios	
Visit 3–4 months	Assess symptoms and compliance	Gastroenterologist and/or dietician; Face-to-face, telephone, or video call
	Serology (IgA-anti TG2)	
	Routine tests (if previously abnormal)	
At 12 months	Assess Weight, symptoms	Physician and/or dietitian Face-to-face, telephone, or video call
	Diet review	
	Celiac serology, routine tests	
	Thyroid function tests	
	Metabolic status	
	Small bowel biopsy (not routinely)	
At 24 months	Symptoms and dietary review	Physician and/or dietitian Face-to-face, telephone, or video call
	Celiac serology	
	Other tests if clinically indicated	
Thereafter every 1–2 years	Assess symptoms	Physician or dietitian Face-to-face, telephone, or video call
	Consider dietary review	
	Celiac serology	
	Thyroid function tests	
	Other tests as clinically indicate	
	Bone densitometry (if abnormal)	

* Consultation with psychosocial professionals should be provided as needed.

## Data Availability

Not applicable.
